# Supplementary material for: A mixed methods survey of social anxiety, anxiety, depression and wig use in alopecia
Source: BMJ Open. 2017 May 4;7(4):e015468. doi: 10.1136/bmjopen-2016-015468 (PMC5566602; doi:10.1136/bmjopen-2016-015468)
Supplement: Author's manuscript [file bmjopen-2016-015468.draft_revisions.pdf]

BMJ Open

A mixed methods survey of social anxiety, anxiety, depression, and wig use in alopecia.

Journal:	*BMJ Open*
Manuscript ID	bmjopen-2016-015468
Article Type:	Research
Date Submitted by the Author:	20-Dec-2016

Complete List of Authors:	Montgomery, Kerry; University of Sheffield, Psychology<br>White, Caroline; University of Manchester, Dermatopharmacology Unit<br>Thompson, Andrew; University of Sheffield, Department of Psychology

**Primary Subject Heading**:	Dermatology
Secondary Subject Heading:	Health services research, Mental health, Public health, Qualitative research
Keywords:	Psychodermatology, wig provision, alopecia

SCHOLARONE™
Manuscripts

For peer review only

A mixed methods survey of social anxiety, anxiety, depression, and wig use in alopecia.

K. Montogmery[1]., C. White[2]., & A.R. Thompson[1]

[1] Department of Psychology, University of Sheffield

[2] Caroline White RN, Research Nurse, University of Manchester, Faculty of Biology, Medicine and Health, Dermatopharmacology Unit. Telephone: 0161 206 0893. Email: caroline.white@manchester.ac.uk

Corresponding author: Kerry Montgomery, Department of Psychology, University of Sheffield, Cathedral Court, 1 Vicar Lane, Sheffield, S1 2LT. UK. Tel: +44 (0)114 2226637. Email: kmontgomery1@sheffield.ac.uk

Conflict of interest disclosure: The authors have no conflicts of interest to disclose

For peer review only

Abstract

Objectives: This study aimed to examine levels of social anxiety, anxiety and depression reported by people with alopecia, and associations with wig use. The study also sought to report on experiences of wearing wigs in social situations, and the financial implications of wearing wigs.

Design: A cross sectional survey was sent by email to the Alopecia UK charity mailing list and advertised on social media.

Participants: Inclusion criteria were a diagnosis of alopecia, aged thirteen or above and sufficient English to complete the survey. Exclusion criteria included; experiencing hair loss as a result of chemotherapy treatment or primary psychological disorder. Participants (N=338) were predominantly female (97.3%), Caucasian (93.5%), aged between 35-54 years (49.4%) and reported a diagnosis of alopecia areata (33.7%).

Main outcome measures: The Social Phobia Inventory was used to measure symptoms of social anxiety, and the Hospital Anxiety and Depression Scale was used to measure symptoms of anxiety and depression. Survey questions were designed to measure the use of wigs. Open-ended questions enabled participants to comment on experiences of wearing wigs.

Results: Clinically significant levels of social anxiety (47.5%), anxiety (35.5%) and depression (27.5%) were reported. Participants who reported worries about not wearing a wig reported significantly higher levels of depression, $t(103) = 3.40$, p = <.001, anxiety, $t(109) = 4.80$, p = <.001, and social anxiety, $t(294)$, = 3.89, p = <.001. Wearing wigs was reported as increasing social confidence; however, the concealment it afforded was also reported as both reducing fear of negative evaluation, but also maintaining anxiety.

Discussion: The relationship between wearing a wig and social confidence is complex. The psychosocial impact of hair loss needs to be considered when making decisions

For peer review only

regarding funding for wig provision. The provision of psychological interventions alongside improved access to wigs is likely to be beneficial for this population.

Strengths and limitations of this study

- This is the first study to examine NHS wig provision and how people living with alopecia finance wigs.
- Validated measures of social anxiety, anxiety and depression were used to determine levels of distress.
- Survey questions investigated the experience of wearing wigs.
- The sample was predominantly female and Caucasian therefore further research is needed to examine experiences of men and cultural differences in alopecia.
- Participants recruited from Alopecia UK may have been experiencing higher levels of distress than the general population having accessed support by the charity.

For peer review only

Introduction

For many people hair is a central aspect of appearance and self-image therefore hair loss can have a negative impact on self-esteem, body image and confidence[1-2]. Whilst hair loss may have few physical health consequences living with a condition that affects appearance can have a significant impact on every day functioning[3-6]. Studies have shown that people living with alopecia are at higher risk of developing depression, anxiety and social phobia than the general population [7-8].

Higher levels of body dissatisfaction and preoccupation with appearance have been reported in men and women living with alopecia, relative to controls [7, 9-10], with 40% of women reporting martial problems as a consequence [3]. Perception of hair loss has a significant impact on quality of life, accounting for 35% of the variance [11].

Despite the psychological consequences of alopecia, access to psychological support within dermatology is limited [12] and currently there are no psychological interventions available to target the specific needs of this population [13]. The need for psychological interventions for alopecia has been emphasised by patients and clinicians [14]. The current study aims to examine levels of clinically significant anxiety, depression and social anxiety in people living with alopecia to determine the psychological impact of hair loss.

Wearing a wig is an important coping strategy for many individuals [15-18] giving people the confidence to return to work and engage in social activity [17]; however, despite this, many people experience difficulties accessing wig prescriptions via the NHS. Some NHS trusts report that alopecia is a 'cosmetic issue' therefore the financial burden of wigs falls with the patient. Further cuts in dermatology could mean more patients have difficulties accessing wigs which may have significant psychosocial consequences for people living with alopecia.

For peer review only

The current mixed methods study aims to examine (i) levels of social anxiety, anxiety and depression reported by people living with alopecia, (ii) associations between wig behaviours and psychosocial distress, and (iii) experiences of wearing wigs in social situations. Given recent changes in NHS wig provision the current study will also investigate how participants access wigs and the financial implications of wearing wigs.

Method

A cross sectional survey incorporating validated measures of psychosocial distress, questions on wig usage, financing wigs, and open ended questions regarding experiences of wearing wigs in social situations was sent by email to the Alopecia UK mailing list, and advertised on social media. Ethical approval for the study was gained from the University of Sheffield Psychology department ethics committee. Inclusion criteria were: a diagnosis of alopecia as a result of a dermatological condition, aged thirteen or above and sufficient English to complete the survey. Exclusion criteria included; experiencing hair loss as a result of chemotherapy treatment or primary psychological disorder (e.g., trichotillomania).

Analysis

Descriptive statistics were used to provide information on (i) demographic and clinical variables, (ii) percentage of people wearing wigs and the situations in which people would choose to wear wigs, (iii) wig funding and (iii) clinically significant levels of social anxiety, depression and anxiety. Associations were tested between demographic variables, wig behaviour (e.g., situations in which people would choose to wear wigs) and psychosocial distress (social anxiety, anxiety and depression) using t tests or correlations as appropriate.

For peer review only

Qualitative responses were analysed using qualitative content analysis [19]. QCA is a systematic text analysis technique which preserves the advantages of quantitative analysis, allowing frequencies of data to be reported whilst also providing a method of interpreting participant experiences of wearing wigs during social situations [19].

An inductive approach to data analysis was used to determine emergent themes generated through examination of participant's responses to specific questions. The data was analysed based on the wording used by participants to describe their experiences, and organised into higher order themes. For example, the response that wigs 'improved confidence' and participants 'felt more able to go out', was coded into the themes 'improved confidence' and 'positive experience of a wearing wig'. Frequency of the occurrence of themes across participants was recorded by the first and second author. Inter-rater reliability (98%) was established by determining any differences in coding between the first and second authors.

Participants

Participants (n =338) were predominantly female (97.3%) and Caucasian (93.5%). The majority of the sample were aged between 35-54 years (49.4%) (13-17 years = 1.5%, 18-25 years = 8%, 26-34 years = 13.6%, 55-64 years = 18.6%, 65 years and over = 8.9%). Eleven different types of alopecia were reported across the sample, the majority of the sample comprised of alopecia areata (33.7%), alopecia universalis (31.4%), and alopecia totalis (17.5%), other subtypes accounted for 2% of the sample or less. Of the respondents 10.7% were receiving medical treatment for hair loss including steroid injections, spironolactone, and topical treatments.

The most common type of wigs worn by participants were acrylic (acrylic monofilament 39.3%, acrylic lace front 27.5% and acrylic wefted wig 14.8). The majority of participants

For peer review only

did not obtain NHS wig prescriptions (50.6%) with 11.2% of participants been told they were not eligible and 22.5% had never enquired about it. Of participants who claimed NHS wig prescriptions (46.7%), 11.5% claimed one per year, 26 % claimed two per year and 8.6% claimed more than two each year.

Measures

Social anxiety

The Social phobia inventory [20] is a 17 item self-report questionnaire to measure various elements of social anxiety (Fear in social situations, avoidance of social situations/ performances and physiological symptoms in social situations). A score of 19 or above indicates symptoms of social anxiety with 79% diagnostic accuracy [20].

Depression

The 9-item self-report Patient health questionnaire [21] was used to measure symptoms of depression experienced over the last two weeks. A cut-off score of ≥ 10 was used to identify individuals experiencing depression (88% specificity 88% sensitivity) [22].

Anxiety

The Generalised anxiety disorder questionnaire [23] is a 7 item self-report scale to measure generalized anxiety symptoms over the last two weeks. A cut off score of ≥ 8 was used to identify individuals experiencing depression [24].

In addition, a number of survey questions were included in which participants were asked to report on the situations in which they would choose to wear a wig (e.g., social situations, dating, work/university/school), and if they had any worries about financing or

For peer review only

not wearing wigs. Open ended questions were asked to elicit experiences of wearing wigs in social situations.

Results

Psychological distress

Clinically significant levels of anxiety (score ≥ 8) were reported by 35.5% of participants, with 40.5% reporting no symptoms, 14.5% reporting mild symptoms (score 7-9), 13.5% reporting moderate symptoms and 15% reporting severe symptoms of anxiety. Clinically significant levels of depression (score ≥ 10) were reported by 27.5% of the sample, with 40.2% reporting no symptoms, 25.7% reporting mild symptoms (score 5-9), 13.4% reporting moderate symptoms, 7.5% reporting severe symptoms and 6.6% reporting severe symptoms of depression. Clinically significant symptoms of social anxiety (score ≥ 19) were reported by 47.5% of the sample, with 23.4% reporting mild symptoms, 11.1% reporting moderate symptoms, 7.2% reporting severe symptoms and 6.6% reporting very severe symptoms. Age was significantly negatively correlated with anxiety, $r(337) = -.12$, $p = .028$, and social anxiety, $r(337) = -.12$, $p = .027$. There was no significant correlation between age and depression, $r(315)$, = -.09, $p = .097$.

Participants reporting worries about not wearing a wig reported significantly higher levels of depression, $t(103) = 3.40$, $p = <.001$, anxiety, $t(109) = 4.80$, $p = <.001$, and social anxiety, $t(294)$, = 3.89, $p = <.001$.

Wig use

Participants were asked when they wear a wig across a number of everyday activities (Table 1), the majority (insert percentage) of participants reported wearing a wig for work

For peer review only

and for socialising, and 66.3% of respondents reported they would not feel confident leaving the house without a wig.

Of the participants who purchased wigs via NHS prescription, 28.1% reported that they would be unable to afford their wig privately and 65.1% of the sample reported worry about affording new wigs. Age was significantly correlated with concerns about affording new wigs, $r(319) = .21$, $p = 001$.

Qualitative findings

A summary of responses to open-ended survey responses is reported below.

Has wearing a wig had any impact on confidence during social situations?

Three hundred and thirteen participants responded to the question on the impact of wigs on confidence in social situations with 26% reporting a positive impact related to the reduced likelihood of comments regarding hair loss (23%), and feeling more confident going out in public (32%).

> *"Interacting with people who I've never met before, without a wig they may wonder why I have no hair, it is not, the norm to have patchy bald head. Hate the idea people will talk about me, or feel I have a more serious illness"*

> *"At different times in my life a wig has been a survival tool for me"*

Participants also reported that wearing a wig could have a negative impact on confidence during social situations (43%) these responses were categorised into two main themes, worries about others knowing it was a wig (47%) and concerns about the wig coming off or discomfort (39%). Wearing a wig also led to reduced activity and a

For peer review only

negative impact on activity (41%) in particular sports were avoided due to concerns about having to take off the wig.

> *"Even 10 years on, I still wonder if people can tell I wear a wig and especially when I meet new people. It has stopped me going to places or trying new activities"*

Difficulty adjusting to a change in appearance when wearing a wig was reported with participants describing feeling disconnected from their appearance in a wig.

> *"No matter how attractive I look in a wig I feel like a sham. I feel the real bald me is not attractive at all"*

For some participants (7.8%) wearing a wig had no impact on their confidence, which was due to positive experiences of wearing a wig. Other responses were not coded as participants had simply responded 'no' which was interpreted as 'no impact' but it was unclear if this was related to a positive or negative impact of wigs.

How has wearing a wig affected your everyday life?

Of two hundred and ninety-nine responses, 23% of participant's reported that wearing a wig improved their confidence/self-esteem in everyday life.

> *"It's improved my confidence as I avoided harsh overhead lights in retail stores as it emphasised my thinning hair particularly distressing when I saw my reflection in a mirror. Now I can walk past a mirror with harsh lighting overhead and check my hair and smile!"*

> *"It's given me a lot more confidence in just leaving the house and being in front of other people."*

For peer review only

Negative views of appearance without a wig were reported (14%).

"Don't like anyone seeing me without it, feel naked."

"I feel a lot less feminine without hair"

Participants reported negative reactions from others if they were not wearing a wig (9.6%) suggesting wigs were used as a coping strategy to manage negative reactions from others.

"It is an uncomfortable but manageable part of my life. It means that I don't get as many pitying glances from strangers."

"I feel more confident to go out with my children, it becomes incredibly tiring been stared at and shouted at if I ever left the house without my wig on."

Overall, 46.6% of participants reported that wearing a wig had a positive impact on their everyday life, reducing the likelihood of negative reactions whilst improving confidence in regards to appearance.

Participants also reported problems with wigs which had a negative impact on everyday life (49%; subthemes included: wigs coming off, expense, quality and fitting).

"Wearing a wig is a way of life to me as I've never had hair, but always dreamed of being normal. Not worrying if it's going to fall off or move"

"Every time I step outside my front door I think about weather conditions, who I might meet, if anyone is going to stand too close to me, what they will see, what they will think."

In addition, wearing wigs reduced social activity (33%) in particular, dating, meeting new people and exercise were situations in which participants experienced problems.

For peer review only

“It’s depressing every morning having to wake up and put my wig on. When I sleep with someone new in my room I don’t take my wig off “

“Constant worry that someone/child will randomly pull the hair, or know it is a wig and make comments. Still anxious when out, but at least I now leave the house.”

“Going swimming is difficult I don't like swimming hats, and use an old wig, but can't dive in a pool or swim under water with a wig on, constantly scared of losing wig in water, but love to swim.”

Participants reported feeling more self-conscious and less confident wearing wigs (17%), using words such as shame, anxiety and freak. Concerns about others finding out they were wearing a wig were also raised (22%)

“The NHS wigs look like wigs so I always imagine people are saying “Look at her wearing that stupid wig ". I only go out if absolutely necessary and avoid public places as often as I can. I FEEL LIKE A FREAK”

“I've had bad experiences at work and socially where someone took my wig for a bet/ cos they were drunk”

Worries about not wearing a wig

One hundred and thirty-five participants reported worries about not wearing a wig with 48.9% of participants reporting concerns about the reactions of others when not wearing a wig (staring 42%, comments 18%). This suggests wearing a wig is a coping strategy to manage negative reactions from others.

“People as a rule have hair, why would I want people to stare and comment?”

For peer review only

Specific concerns about not wearing a wig included worries others would assume they had cancer (n = 13).

"People assume I have cancer. I look strange"

Self-conscious emotions and negative self-appraisals of appearance also led to worries about not wearing a wig (19%).

"I am worried people will see how ugly I really am if they see me bald"

Discussion

Social anxiety was commonly reported by the participants in this study and with 47.5% indicating clinically significant levels of social anxiety, it is important to identify potential coping strategies to help people in managing their distress. Specific psychological support may be beneficial. For some people living with alopecia, this study clearly shows that wearing a wig is an important coping strategy, helping people to manage negative reactions from others and improving confidence to engage in social activity. However, despite wigs being viewed as an important coping strategy only 46.7% of the sample accessed NHS wig prescriptions, with some NHS trusts failing to provide any wig provision for patients with alopecia.

Worry about not wearing a wig was related to higher levels of depression, anxiety and social anxiety and with 65% of participants reporting worry about affording new wigs, it suggests the financial burden of affording wigs could lead to additional distress in patients already reporting significant levels of social anxiety, depression and anxiety. This finding has important implications in the current economic climate and suggests changes in NHS wig provision could have negative psychosocial consequences should people with alopecia no longer be able to access wig prescriptions. In the current sample age was significantly negatively correlated with social anxiety and anxiety, suggesting

For peer review only

that younger people living with alopecia may experience higher anxiety; however, this relationship was not significant for depression.

The current findings indicate that people living with alopecia face a difficult choice, negative reactions as a result of hair loss being visible, or negative reactions a result of wearing a wig. Managing the noticeability of wigs appeared to lead to significant negative interpersonal consequences, including avoidance of social situations and sports. Despite generating anxiety and causing discomfort, participants were still choosing to wear wigs suggesting wigs were seen as the 'lesser of two evils' given worries about reactions of others to hair loss.

Critical self-appraisals of appearance without a wig ('freak, alien, ugly') appeared to be a contributing factor to wearing a wig, as wigs improved confidence in social situations through reducing appearance concern. Hair loss was viewed as a negative attribute with participants reporting experiences of stigma including comments and staring, and perception of hair loss as a sign of illness. Hair loss is reported as confirmation of identity for cancer patients [25] which has social implications for people living with alopecia, in that they are often mis-identified as 'ill'.

Negative reactions from others represent threats to an individual's desire to be valued and accepted by others [26]. Reactions to social rejection may be prosocial, involving an increased desire to be socially accepted; antisocial, whereby the individual attempts to defend themselves or express anger following rejection, and avoidant [26], in which an individual may seek to avoid further rejection which could involve reduced social engagement. Wearing a wig may therefore be seen as a strategy to increase social acceptance and to avoid rejection. Indeed, Goffman's seminal work on stigma [27] suggests that individuals may choose to conceal attributes they believe will be

For peer review only

discredited by others, in this case hair loss; however, the consequence of this is continued concealment and anxiety about being ‘found out’.

The current findings suggest that the relationship between wigs and social confidence is complex and wigs alone do not appear to reduce social distress for a large number of patients. Poor quality wigs in particular were highlighted as a source of anxiety in social situations, through fears of hair loss being exposed. Findings suggest that improvements in wig quality would help participants to feel more confident in social interaction. However, for many better quality wigs may not be a financially viable option, and with some NHS trusts not providing wig provision this leaves people in an unfortunate position.

There are several limitations of the current study which should be noted. Firstly, the sample is predominantly female and Caucasian therefore further research is needed to explore the experiences of men and cultural differences in experiences of alopecia. Second, a number of responses could not be coded into themes as the responses did not relate to the question suggesting some participants may have experienced problems interpreting the questions. Third, participants were recruited from Alopecia UK, therefore some participants may have been experiencing higher levels of distress than the general population having accessed support via the charity.

The current findings have important implications when providing support to people living with alopecia. The findings suggest that psychological interventions, particularly those targeting self-conscious emotions such as shame and social anxiety would be beneficial for people living with alopecia alongside wig provision. To date, there are no studies examining the effectiveness of psychological interventions specific to people living with alopecia; however, approaches such as cognitive behavioural therapy and compassion

For peer review only

focused therapy may be beneficial in targeting processes underlying social anxiety. Attentional training techniques such as mindfulness may also be beneficial to reduce pre-occupation with appearance related thoughts [28].

Wigs appear to be much more than a ‘cosmetic’ tool, and are used to reduce potential experiences of stigmatisation. Given the significant negative psychosocial consequences that could arise should wigs no longer be an option the current findings should be considered when funding decisions are made regarding wig provision for patients with alopecia

Acknowledgements

The authors would like to thank alopecia UK for their support in recruitment for this study.

Contributors AT and KM developed the protocol for this study. All authors have contributed to the preparation of this manuscript. KM carried out data analysis for quantitative data and KM and CW analysed qualitative data to ensure reliability of coding. All data was checked by AT.

Funding This work was supported by the Economic and Social Research Council Grant number ES/J00215/1

Competing interests None declared.

Ethics approval Department of Psychology ethics committee, University of Sheffield

Data sharing statement The protocol and data files are available from the corresponding author on request.

For peer review only

References

1. Thompson AR. Skin conditions. *Encyclopaedia of Body Image and Human Appearance*; 738-744.
2. Tucker P. Bald is beautiful? The psychosocial impact of alopecia areata. J Health Psychol. 2009;**14**:142-51. doi: 10.1177/1359105308097954
3. Hunt N, McHale SU. Reported experiences of persons with alopecia areata. Journal of Loss and Trauma. 2004;**10**:33-50. doi: 10.1080/15325020490890633
4. Hunt N, McHale S. The psychological impact of alopecia. *BMJ*. 2005; **20**;7522:951.
5. Rumsey N, Harcourt D. Body image and disfigurement: issues and interventions. *Body image*. 2004;**1**(1):83-97. doi: 10.1016/S1740-1445(03)00005-6
6. Thompson A, Kent G. Adjusting to disfigurement: processes involved in dealing with being visibly different. *Clin Psychol Rev*. 2001;**21**(5):663-82. doi: 10.1016/S0272-7358(00)00056-8
7. Ruiz-Doblado S, Carrizosa A, García-Hernández MJ. Alopecia areata: psychiatric comorbidity and adjustment to illness. *Int J Dermatol*. 2003;**42**(6):434-7. doi: 10.1046/j.1365-4362.2003.01340.x
8. Koo JY, Shellow WV, Hallman CP, Edwards JE. Alopecia areata and increased prevalence of psychiatric disorders. *Int J Dermatol*. 1994;**33**(12):849-50. doi: 10.1111/j.1365-4362.1994.tb01018.x
9. Cash TF. The psychological effects of androgenetic alopecia in men. *J Am. Acad. Dermatol*. 1992;**26**(6):926-31. doi: 10.1016/0190-9622(92)70134-2
10. Cash TF, Price VH, Savin RC. Psychological effects of androgenetic alopecia on women: comparisons with balding men and with female control subjects. *J Am. Acad. Dermatol*. 1993;**29**(4):568-75. doi: 10.1016/0190-9622(93)70223-G
11. Chiang YZ, Bundy C, Griffiths CE, Paus R, Harries MJ. The role of beliefs: lessons from a pilot study on illness perception, psychological distress and quality of life in patients with primary cicatricial alopecia. *Brit J Dermatol*. 2015;**172**(1):130-7. doi: 10.1111/bjd.13259
12. Bewley A, Affleck A, Bundy C, Higgins E, McBride S. Psychodermatology services guidance: The report of the British Association of Dermatologists' Psychodermatology Working Party. *Brit J Dermatol*. 2013;**168**:1149-50. doi: 10.1111/bjd.12330
13. Lavda AC, Webb T, Thompson AR. A meta-analysis of the effectiveness of psychological interventions for adults with skin conditions. *Brit J Dermatol*. 2012;**167**:970-9. doi: 10.1111/j.1365-2133.2012.11183.x
14. Macbeth AE, Tomlinson J, Messenger AG, Moore-Millar K, Michaelides C, Shipman AR, Kassim JM, Brockley JR, Szczecinska W, Farrant P, Robinson R. Establishing and Prioritising Research Questions for the Treatment of Alopecia Areata: The Alopecia Areata Priority Setting Partnership. *Brit J Dermatol*. 2016; Oct 1. doi: 10.1111/bjd.15099

For peer review only

15. Messenger AG, McKillop J, Farrant P, McDonagh AJ, Sladden M. British Association of Dermatologists' guidelines for the management of alopecia areata 2012. *Brit J Dermatol*. 2012;**166**(5):916-26. doi: 10.1111/j.1365-2133.2012.10955.x

16. Wiggins S, Moore-Millar K, Thomson A. Can you pull it off? Appearance modifying behaviours adopted by wig users with alopecia in social interactions. *Body image*. 2014;**11**(2):156-66. doi: 10.1016/j.bodyim.2014.01.004

17. Welsh N, Guy A. The lived experience of alopecia areata: a qualitative study. *Body Image*. 2009;**6**(3):194-200. 10.1016/j.bodyim.2009.03.004

18. Cash TF. The psychology of hair loss and its implications for patient care. *Clin Dermatol*. 2001;**19**(2):161-6. doi: 10.1016/S0738-081X(00)00127-9

19. Mayring P. Qualitative content analysis. *Qualitative Social Research*. 2000; **1**(2). Available from: www.qualitative-research.net/index.php/fqs/article/view/1089/2385 (Accessed 29th August 2016)

20. Connor KM, Davidson JR, Churchill LE, Sherwood A, Weisler RH, Foa E. Psychometric properties of the social phobia inventory (SPIN). *Brit J Psychiat*. 2000;**176**(4):379-86. doi: 10.1192/bjp.176.4.379

21. Spitzer RL, Kroenke K, Williams JB, Patient Health Questionnaire Primary Care Study Group. Validation and utility of a self-report version of PRIME-MD: the PHQ primary care study. *Jama.* 1999;**282**(18):1737-44. doi: 10.1001/jama.282.18.1737

22. Kroenke K, Spitzer RL, Williams JB. The Phq-9. *J Gen Intern Med*. 2001;**16**(9):606-13. doi: 10.1046/j.1525-1497.2001.016009606.x

23. Spitzer RL, Kroenke K, Williams JB, Löwe B. A brief measure for assessing generalized anxiety disorder: the GAD-7. *Archives of Internal Medicine.* 2006;**166**(10):1092-7. doi: /10.1001archinte.166.10.1092

24. Kroenke K, Spitzer RL, Williams JBW, Monahan PO, Löwe B. Anxiety disorders in primary care: prevalence, impairment, comorbidity, and detection. *Ann Intern Med*. 2007;**146**(5):317–25. doi: 10.7326/0003-4819-146-5-200703060-00004

25. Rosman S. Cancer and stigma: experience of patients with chemotherapy-induced alopecia. *Patient Edu Couns*. 2004;**52**(3):333-9. doi: 10.1016/S0738-3991(03)00040-5

26. Smart Richman L, Leary MR. Reactions to discrimination, stigmatization, ostracism, and other forms of interpersonal rejection: a multimotive model. Psychol Rev. 2009;**116**(2):365. doi: 10.1037/a0015250

27. Goffman E. *Stigma: Notes on the management of spoiled identity*. Simon and Schuster. 2009.

28. Clarke A, Thompson AR, Jenkinson E, Rumsey N, Newell R. *CBT for appearance anxiety: psychosocial interventions for anxiety due to visible difference*. John Wiley & Sons. 2013.

For peer review only

Table 1. Situations in which people would wear a wig

Situations when you would wear a wig	% of people wearing a wig
Meeting new people	78.4
Dating	61.2
Socialising	86.7
Work/school/university	76.3
All the time	55.9

For peer review only

STROBE Statement

	Item No	Recommendation	Page Number
**Title and abstract**	1	(*a*) Indicate the study's design with a commonly used term in the title or the abstract	1
		(*b*) Provide in the abstract an informative and balanced summary of what was done and what was found	2
**Introduction**			
Background/rationale	2	Explain the scientific background and rationale for the investigation being reported	4-5
Objectives	3	State specific objectives, including any prespecified hypotheses	4-5
**Methods**			
Study design	4	Present key elements of study design early in the paper	5
Setting	5	Describe the setting, locations, and relevant dates, including periods of recruitment, exposure, follow-up, and data collection	5
Participants	6	(*a*) *Cohort study*—Give the eligibility criteria, and the sources and methods of selection of participants. Describe methods of follow-up<br>*Case-control study*—Give the eligibility criteria, and the sources and methods of case ascertainment and control selection. Give the rationale for the choice of cases and controls<br>*Cross-sectional study*—Give the eligibility criteria, and the sources and methods of selection of participants	Cross-sectional study<br>5 (paragraph 1) further information on participant characteristics on p. 6
		(*b*) *Cohort study*—For matched studies, give matching criteria and number of exposed and unexposed<br>*Case-control study*—For matched studies, give matching criteria and the number of controls per case	
Variables	7	Clearly define all outcomes, exposures, predictors, potential confounders, and effect modifiers. Give diagnostic criteria, if applicable	7
Data sources/ measurement	8*	For each variable of interest, give sources of data and details of methods of assessment (measurement). Describe comparability of assessment methods if there is more than one group	7
Bias	9	Describe any efforts to address potential sources of bias	Cross-sectional study. Recruitment via charity discussed in limitations p.15
Study size	10	Explain how the study size was arrived at	6

For peer review only - http://bmjopen.bmj.com/site/about/guidelines.xhtml
For peer review only

Quantitative variables	11	Explain how quantitative variables were handled in the analyses. If applicable, describe which groupings were chosen and why	5-6
Statistical methods	12	(*a*) Describe all statistical methods, including those used to control for confounding	5-6
		(*b*) Describe any methods used to examine subgroups and interactions	n/a
		(*c*) Explain how missing data were addressed	n/a
		(*d*) *Cohort study*—If applicable, explain how loss to follow-up was addressed<br>*Case-control study*—If applicable, explain how matching of cases and controls was addressed<br>*Cross-sectional study*—If applicable, describe analytical methods taking account of sampling strategy	All responses complete
		(*e*) Describe any sensitivity analyses	n/a

Continued on next page

For peer review only - http://bmjopen.bmj.com/site/about/guidelines.xhtml

**Results**			**Page**
Participants	13*	(a) Report numbers of individuals at each stage of study—eg numbers potentially eligible, examined for eligibility, confirmed eligible, included in the study, completing follow-up, and analysed	6
		(b) Give reasons for non-participation at each stage	n/a
		(c) Consider use of a flow diagram	n/a
Descriptive data	14*	(a) Give characteristics of study participants (eg demographic, clinical, social) and information on exposures and potential confounders	6
		(b) Indicate number of participants with missing data for each variable of interest	n/a
		(c) *Cohort study*—Summarise follow-up time (eg, average and total amount)	n/a
Outcome data	15*	*Cohort study*—Report numbers of outcome events or summary measures over time	n/a
		Case-control study—Report numbers in each exposure category, or summary measures of exposure	n/a
		Cross-sectional study—Report numbers of outcome events or summary measures	6-7
Main results	16	(*a*) Give unadjusted estimates and, if applicable, confounder-adjusted estimates and their precision (eg, 95% confidence interval). Make clear which confounders were adjusted for and why they were included	n/a
		(*b*) Report category boundaries when continuous variables were categorized	7
		(*c*) If relevant, consider translating estimates of relative risk into absolute risk for a meaningful time period	n/a
Other analyses	17	Report other analyses done—eg analyses of subgroups and interactions, and sensitivity analyses	n/a
**Discussion**			
Key results	18	Summarise key results with reference to study objectives	13-15
Limitations	19	Discuss limitations of the study, taking into account sources of potential bias or imprecision. Discuss both direction and magnitude of any potential bias	15
Interpretation	20	Give a cautious overall interpretation of results considering objectives, limitations, multiplicity of analyses, results from similar studies, and other relevant evidence	16
Generalisability	21	Discuss the generalisability (external validity) of the study results	15
**Other information**			
Funding	22	Give the source of funding and the role of the funders for the present study and, if applicable, for the original study on which the present article is based	n/a

*Give information separately for cases and controls in case-control studies and, if applicable, for exposed and unexposed groups in cohort and cross-sectional studies.

Note: An Explanation and Elaboration article discusses each checklist item and gives methodological background and published examples of transparent reporting. The STROBE checklist is best used in conjunction with this article (freely available on the Web sites of PLoS Medicine at

For peer review only

http://www.plosmedicine.org/, Annals of Internal Medicine at http://www.annals.org/, and Epidemiology at http://www.epidem.com/). Information on the STROBE Initiative is available at www.strobe-statement.org.

For peer review only

BMJ Open

A mixed methods survey of social anxiety, anxiety, depression, and wig use in alopecia.

Journal:	*BMJ Open*
Manuscript ID	bmjopen-2016-015468.R1
Article Type:	Research
Date Submitted by the Author:	03-Mar-2017
Complete List of Authors:	Montgomery, Kerry; University of Sheffield, Psychology<br>White, Caroline; University of Manchester, Dermatopharmacology Unit<br>Thompson, Andrew; University of Sheffield, Department of Psychology
**Primary Subject Heading**:	Dermatology
Secondary Subject Heading:	Health services research, Mental health, Public health, Qualitative research
Keywords:	Psychodermatology, wig provision, alopecia

SCHOLARONE™ Manuscripts

For peer review only

A mixed methods survey of social anxiety, anxiety, depression, and wig use in alopecia.

K. Montogmery[1]., C. White[2]., & A.R. Thompson[1]

[1] Department of Psychology, University of Sheffield

[2] Caroline White RN, Research Nurse, University of Manchester, Faculty of Biology, Medicine and Health, Dermatopharmacology Unit. Telephone: 0161 206 0893. Email: caroline.white@manchester.ac.uk

Corresponding author: Kerry Montgomery, Department of Psychology, University of Sheffield, Cathedral Court, 1 Vicar Lane, Sheffield, S1 2LT. UK. Tel: +44 (0)114 2226637. Email: kmontgomery1@sheffield.ac.uk

Conflict of interest disclosure: The authors have no conflicts of interest to disclose

For peer review only

Abstract

Objectives: This study aimed to examine levels of social anxiety, anxiety and depression reported by people with alopecia, and associations with wig use. The study also sought to report on experiences of wearing wigs in social situations, and the financial implications of wearing wigs.

Design: A cross sectional survey was sent by email to the Alopecia UK charity mailing list and advertised on social media.

Participants: Inclusion criteria were a diagnosis of alopecia, aged thirteen or above and sufficient English to complete the survey. Exclusion criteria included; experiencing hair loss as a result of chemotherapy treatment or primary psychological disorder. Participants (N=338) were predominantly female (97.3%), Caucasian (93.5%), aged between 35-54 years (49.4%) and reported a diagnosis of alopecia areata (82.6%).

Main outcome measures: The Social Phobia Inventory was used to measure symptoms of social anxiety, and the Hospital Anxiety and Depression Scale was used to measure symptoms of anxiety and depression. Survey questions were designed to measure the use of wigs. Open-ended questions enabled participants to comment on experiences of wearing wigs.

Results: Clinically significant levels of social anxiety (47.5%), anxiety (35.5%) and depression (29%) were reported. Participants who reported worries about not wearing a wig reported significantly higher levels of depression, $t(103) = 3.40$, p = <.001, anxiety, $t(109) = 4.80$, p = <.001, and social anxiety, $t(294)$, = 3.89, p = <.001. Wearing wigs was reported as increasing social confidence; however, the concealment it afforded was also reported as both reducing fear of negative evaluation, but also maintaining anxiety.

For peer review only

Discussion: The relationship between wearing a wig and social confidence is complex. The psychosocial impact of hair loss needs to be considered when making decisions regarding funding for wig provision.

Strengths and limitations of this study

- This is the first study to examine National Health Service wig provision and how people living with alopecia finance wigs.
- Validated measures of social anxiety, anxiety and depression were used to determine levels of distress.
- Survey questions investigated the experience of wearing wigs.
- The sample was predominantly female and Caucasian therefore further research is needed to examine experiences of men and cultural differences in alopecia.
- Participants recruited from Alopecia UK may have been experiencing higher levels of distress than the general population having accessed support by the charity.

For peer review only

Introduction

For many people hair is a central aspect of appearance and self-image therefore hair loss can have a negative impact on self-esteem, body image and confidence [1-2]. Whilst hair loss may have few physical health consequences living with a condition that affects appearance can have a significant impact on every day functioning [3-6]. Studies have shown that people living with alopecia are at higher risk of developing depression, anxiety and social phobia than the general population [7-8].

Higher levels of body dissatisfaction and preoccupation with appearance have been reported in men and women living with alopecia, relative to controls [7, 9-10], with 40% of women reporting marital problems as a consequence [3]. Perception of hair loss has a significant impact on quality of life, accounting for 35% of the variance [11].

Wearing a wig to conceal hair loss is an important coping strategy for many individuals [12-15] giving people the confidence to return to work and engage in social activity [14]. We have known since the time of Goffman's seminal work on stigmatisation [16] that people may choose to conceal attributes they believe will be discredited by others. Recent studies indicate that alopecia areata can be associated with higher levels of perceived stigmatisation than concealable conditions such as mental health conditions [17]. Wearing a wig might reduce an individuals' perception of a discrepancy between cultural norms regarding the appearance of the hair and their own appearance. However, wearing a wig might not be an ideal solution as the fear of being 'discredited' should the wig be noticed remains [16].

Despite wigs being an important coping strategy for people living with alopecia many people experience difficulties accessing wig prescriptions via the National Health Service (NHS). Some NHS trusts report that alopecia is a 'cosmetic issue' therefore the financial

For peer review only

burden of wigs falls with the patient. Further cuts in dermatology could mean more patients have difficulties accessing wigs which may have significant psychosocial consequences for people living with alopecia. Access to psychological support within dermatology is limited [18] and currently there are no psychological interventions available to target the specific needs of this population [19]. The need for psychological interventions for alopecia has been emphasised by patients and clinicians [20].

The current mixed methods study aims to examine (i) levels of social anxiety, anxiety and depression reported by people living with alopecia, (ii) associations between wig behaviours and psychosocial distress, and (iii) experiences of wearing wigs in social situations. The current study will also investigate how participants access wigs and the financial implications of wearing wigs.

Method

A cross sectional survey incorporating validated measures of social anxiety, depression and anxiety, questions on wig usage, financing wigs, and open ended questions regarding experiences of wearing wigs in social situations was sent by email to the Alopecia UK mailing list, and advertised on social media. Ethical approval for the study was gained from the University of Sheffield Psychology department ethics committee. Inclusion criteria were: a diagnosis of alopecia as a result of a dermatological condition, aged thirteen or above and sufficient English to complete the survey. Participants aged under 16 years of age were advised to complete the questionnaire with a parent or guardian. Exclusion criteria included; experiencing hair loss as a result of chemotherapy treatment or primary psychological disorder (e.g., trichotillomania).

Measures

Social anxiety

For peer review only

The Social phobia inventory (SPIN) [21] is a 17 item self-report questionnaire to measure various elements of social anxiety (Fear in social situations, avoidance of social situations/ performances and physiological symptoms in social situations). Connor et al [21] reported a mean score of 41 on the SPIN for individuals with social phobia, compared to a score of 12 in a non-psychiatric population. A score of 19 or above indicates symptoms of social anxiety with 79% diagnostic accuracy [21].

Depression

The 9-item self-report Patient Health Questionnaire [22] was used to measure symptoms of depression experienced over the last two weeks. A mean score of three has been reported by healthy controls, in comparison to a score of 17 in people diagnosed with depression [22]. A cut-off score of ≥ 10 was used to identify individuals experiencing depression (88% specificity 88% sensitivity) [23].

Anxiety

The Generalised Anxiety Disorder Questionnaire [24] is a 7 item self-report scale to measure generalized anxiety symptoms over the last two weeks. A mean score of 4.9 has been reported in healthy controls, in comparison to a score of 14 in people living with generalised anxiety [24]. A cut off score of ≥ 8 was used to identify individuals experiencing depression [25].

Survey questions

A number of survey questions were included in which participants were asked to report on the situations in which they would choose to wear a wig (e.g., social situations, dating, work/university/school), and if they had any worries about financing or not wearing wigs. Open ended questions were asked to elicit experiences of wearing wigs in social

For peer review only

situations. Questions included: Has wearing a wig had any impact on your confidence during social situations? Do you have any worries about not wearing a wig? and, how has wearing a wig affected your everyday life?

Analysis

Statistical analysis was carried out using SPSS version 22 [26]. Descriptive statistics were used to provide information on (i) demographic and clinical variables, (ii) percentage of people wearing wigs and the situations in which people would choose to wear wigs, (iii) wig funding and (iii) clinically significant levels of social anxiety, depression and anxiety. Associations were tested between demographic variables, wig behaviour (e.g., situations in which people would choose to wear wigs) and social anxiety, depression and anxiety using independent samples t tests and Pearson correlation as appropriate. An alpha level of .05 was used for all statistical tests.

Qualitative responses were analysed using qualitative content analysis [27]. QCA is a systematic text analysis technique which preserves the advantages of quantitative analysis, allowing frequencies of data to be reported whilst also providing a method of interpreting participant experiences of wearing wigs during social situations [27].

An inductive approach to data analysis was used to determine emergent themes generated through examination of participant's responses to specific questions. The data was analysed based on the wording used by participants to describe their experiences, and organised into higher order themes. For example, the response that wigs 'improved confidence' and participants 'felt more able to go out', was coded into the themes 'improved confidence' and 'positive experience of a wearing wig'. Frequency of the occurrence of themes across participants was recorded by the first and second author.

For peer review only

Inter-rater reliability (98%) was established by determining any differences in coding between the first and second authors.

Results

Participants

Participants (N =338) were predominantly female (97.3%) and Caucasian (93.5%). All participants lived in the UK. The majority of the sample were aged between 35-54 years (49.4%) (13-17 years = 1.5%, 18-25 years = 8%, 26-34 years = 13.6%, 55-64 years = 18.6%, 65 years and over = 8.9%). Eleven different types of alopecia were reported across the sample (Table 1) and the majority of the sample comprised of alopecia areata (82.6%). Of the respondents 10.7% were receiving medical treatment for hair loss including steroid injections, spironolactone, and topical treatments.

The majority of participants chose to wear wigs most or all of the time (76%) (Table 2). The most common type of wigs worn by participants were acrylic (acrylic monofilament 39.3%, acrylic lace front 27.5% and acrylic wefted wig 14.8). The majority of participants did not obtain NHS wig prescriptions (50.6%) with 11.2% of participants been told they were not eligible and 22.5% had never enquired about it. Of participants who claimed NHS wig prescriptions (46.1%), 11.5% claimed one per year, 26 % claimed two per year and 8.6% claimed more than two each year.

Social anxiety, anxiety and depression

Clinically significant levels of anxiety (score ≥ 8) were reported by 35.5% of participants, with 40.5% reporting no symptoms, 14.5% reporting mild symptoms (score 7-9), 13.5% reporting moderate symptoms and 15% reporting severe symptoms of anxiety. Clinically significant levels of depression (score ≥ 10) were reported by 29% of the sample, with

For peer review only

43.6% reporting no symptoms, 27.6% reporting mild symptoms (score 5-9), 14.3% reporting moderate symptoms (score 10-14), 8.1% reporting moderately severe symptoms and 6.6% reporting severe symptoms of depression. Clinically significant symptoms of social anxiety (score ≥ 19) were reported by 47.5% of the sample, with 23.4% reporting mild symptoms, 11.1% reporting moderate symptoms, 7.2% reporting severe symptoms and 6.6% reporting very severe symptoms. Participants reporting worries about not wearing a wig reported significantly higher levels of depression, $t(103) = 3.40$, $p = <.001$, anxiety, $t(109) = 4.80$, $p = <.001$, and social anxiety, $t(294)$, $= 3.89$, $p = <.001$.

Wig use

Participants were asked when they wear a wig across a number of everyday activities (Table 3). The majority of participants reported wearing a wig to socialise (86.7%), and 66.3% of respondents reported they would not feel confident leaving the house without a wig. Of the participants who purchased wigs via NHS prescription, 28.1% reported that they would be unable to afford their wig privately and 65.1% of the sample reported worry about affording new wigs.

Qualitative findings

A summary of responses to open-ended survey responses is reported below.

Has wearing a wig had any impact on confidence during social situations?

Three hundred and thirteen participants responded to the question on the impact of wigs on confidence in social situations. Of responses, 26% reported a positive impact of wearing a wig. The positive impact was related to the reduced likelihood of comments

For peer review only

regarding hair loss in 23% of participants, and feeling more confident going out in public (32%).

> *“Interacting with people who I've never met before, without a wig they may wonder why I have no hair, it is not, the norm to have patchy bald head. Hate the idea people will talk about me, or feel I have a more serious illness”*

> *“At different times in my life a wig has been a survival tool for me”*

Participants also reported that wearing a wig could have a negative impact on confidence during social situations (43%). These responses were categorised into two main themes: worries about others knowing it was a wig (47%) and concerns about the wig coming off/ discomfort (39%). Wearing a wig also led to reduced activity (41%) in particular sports were avoided due to concerns about having to take off the wig.

> *“Even 10 years on, I still wonder if people can tell I wear a wig and especially when I meet new people. It has stopped me going to places or trying new activities”*

Difficulty adjusting to a change in appearance when wearing a wig was reported with participants describing feeling disconnected from their appearance in a wig.

> *“No matter how attractive I look in a wig I feel like a sham. I feel the real bald me is not attractive at all”*

For some participants (7.8%) wearing a wig had no impact on their confidence, which was due to positive experiences of wearing a wig. Other responses were not coded as participants had simply responded ‘no’ which was interpreted as ‘no impact’ but it was unclear if this was related to a positive or negative impact of wigs.

For peer review only

How has wearing a wig affected your everyday life?

Of two hundred and ninety-nine responses, 23% of participants reported that wearing a wig improved their confidence/self-esteem in everyday life.

> *"It's improved my confidence as I avoided harsh overhead lights in retail stores as it emphasised my thinning hair particularly distressing when I saw my reflection in a mirror. Now I can walk past a mirror with harsh lighting overhead and check my hair and smile!"*

> *"It's given me a lot more confidence in just leaving the house and being in front of other people."*

Negative views of appearance without a wig were reported (14%).

> *"Don't like anyone seeing me without it, feel naked."*

> *"I feel a lot less feminine without hair"*

Participants reported negative reactions from others if they were not wearing a wig (9.6%) suggesting wigs were used as a coping strategy to manage negative reactions.

> *"It is an uncomfortable but manageable part of my life. It means that I don't get as many pitying glances from strangers."*

> *"I feel more confident to go out with my children, it becomes incredibly tiring been stared at and shouted at if I ever left the house without my wig on."*

Overall, 46.6% of participants reported that wearing a wig had a positive impact on their everyday life, reducing the likelihood of negative reactions whilst improving confidence in regards to appearance.

For peer review only

Participants also reported problems with wigs which had a negative impact on everyday life (49%; subthemes included: wigs coming off, expense, quality and fitting).

> *"Wearing a wig is a way of life to me as I've never had hair, but always dreamed of being normal. Not worrying if it's going to fall off or move"*

> *"Every time I step outside my front door I think about weather conditions, who I might meet, if anyone is going to stand too close to me, what they will see, what they will think."*

In addition, wearing wigs reduced social activity (33%) in particular, dating, meeting new people and exercise were situations in which participants experienced problems.

> *"It's depressing every morning having to wake up and put my wig on. When I sleep with someone new in my room I don't take my wig off "*

> *"Constant worry that someone/child will randomly pull the hair, or know it is a wig and make comments. Still anxious when out, but at least I now leave the house."*

> *"Going swimming is difficult I don't like swimming hats, and use an old wig, but can't dive in a pool or swim under water with a wig on, constantly scared of losing wig in water, but love to swim."*

Participants reported feeling more self-conscious about having to wear a wig (17%). Self-consciousness appeared to arise as a result of concerns about others finding out they were wearing a wig (22%).

> "The NHS wigs look like wigs so I always imagine people are saying "Look at her wearing that stupid wig ". I only go out if absolutely necessary and avoid public places as often as I can. I FEEL LIKE A FREAK"

For peer review only

“I've had bad experiences at work and socially where someone took my wig for a bet/ cos they were drunk”

Worries about not wearing a wig

Participants reported worries about not wearing a wig (40.5%) due to the reactions of others to hair loss (staring 42%, comments 18%).

“People as a rule have hair, why would I want people to stare and comment?”

Specific concerns about not wearing a wig included worries others would assume they had cancer (n = 13).

“People assume I have cancer. I look strange”

Self-conscious emotions and negative self-appraisals of appearance also led to worries about not wearing a wig (19%).

“I am worried people will see how ugly I really am if they see me bald”

Discussion

The first aim of the current study was to investigate the prevalence of social anxiety, anxiety and depression in people living with alopecia. Social anxiety was commonly reported by the participants in this study with 47.5% indicating clinically significant levels of social anxiety. Whilst few quantitative studies have examined the prevalence of social anxiety in people living with alopecia specifically; qualitative studies suggest that alopecia can have a significant impact on social functioning [2-4, 13-14]. The prevalence of anxiety and depression reported are consistent with previous studies [2], and highlights the importance of identifying coping strategies to help people living with alopecia to manage psychological distress.

For peer review only

The current study also aimed to examine the relationship between psychological variables and wig usage. Worry about not wearing a wig was related to higher levels of depression, anxiety and social anxiety and with 65% of participants reporting worry about affording new wigs, it suggests the financial burden of affording wigs could lead to additional distress in patients already reporting significant levels of social anxiety, depression and anxiety. This finding has important implications in the current economic climate and suggests changes in NHS wig provision could have negative psychosocial consequences should people with alopecia no longer be able to access wig prescriptions.

For some people living with alopecia, this study clearly shows that wearing a wig is an important coping strategy, helping people to manage negative reactions from others and improving confidence to engage in social activity. However, despite wigs being viewed as an important coping strategy only 46.7% of participants who chose to wear wigs accessed NHS wig prescriptions, with some NHS trusts failing to provide any wig provision for patients with alopecia.

The final aim of the current study was to explore participants experiences of wearing wigs in social situations. The current findings suggest that the relationship between wigs and social confidence is complex and wigs alone do not appear to reduce social distress for a large number of patients. The current findings indicate that people living with alopecia face a difficult choice in social situations, negative reactions as a result of hair loss being visible, or negative reactions a result of wearing a wig. Managing the noticeability of wigs appeared to lead to significant negative interpersonal consequences, including avoidance of social situations and exercise. Despite generating anxiety and causing discomfort, participants were still choosing to wear wigs suggesting wigs were seen as the ‘lesser of two evils’ given worries about reactions of others to hair loss.

For peer review only

These findings are consistent with previous studies and suggest that wearing a wig does not signify an end point to managing social distress for people living with alopecia[13-14].

Critical self-appraisals of appearance without a wig (‘freak, alien, ugly’) appeared to be a contributing factor to wearing a wig, as wigs improved confidence in social situations through reducing appearance concern. Hair loss was viewed as a negative attribute with participants reporting experiences of stigma including comments and staring, and perception of hair loss as a sign of illness. Hair loss is reported as confirmation of identity for cancer patients [28] which has social implications for people living with alopecia, in that they are often mis-identified as ‘ill’.

Negative reactions from others represent threats to an individual’s desire to be valued and accepted by others [16]. Reactions to social rejection may be prosocial, involving an increased desire to be socially accepted; antisocial, whereby the individual attempts to defend themselves or express anger following rejection, and avoidant [29], in which an individual may seek to avoid further rejection which could involve reduced social engagement. Wearing a wig may therefore be seen as a strategy to increase social acceptance and to avoid rejection. Indeed, Goffman’s seminal work on stigma [16] suggests that individuals may choose to conceal attributes they believe will be discredited by others, in this case hair loss; however, the consequence of this is continued concealment and anxiety about being ‘found out’.

There are several limitations of the current study which should be noted. Firstly, the sample is predominantly female and Caucasian therefore further research is needed to explore the experiences of men and cultural differences in experiences of alopecia. Second, a number of responses could not be coded into themes as the responses did not relate to the question suggesting some participants may have experienced problems

For peer review only

interpreting the questions. Third, participants were recruited from Alopecia UK, therefore some participants may have been experiencing higher levels of distress than the general population having accessed support via the charity. Finally, given that participants were recruited from the Alopecia UK mailing list and social media it is not possible to ascertain a response rate in the current study.

The current findings have important implications when providing support to people living with alopecia and suggest that wigs appear to be much more than a 'cosmetic' tool, and are used to reduce potential experiences of stigmatisation and improve social confidence. Given the significant negative social consequences that could arise should wigs no longer be an option, the current findings should be considered when funding decisions are made regarding wig provision for patients with alopecia. Negative experiences of wearing wigs are related to fears of the wig being noticed and hair loss being discovered; therefore, psychological interventions, particularly those targeting self-conscious emotions such as shame and social anxiety would be beneficial for people living with alopecia alongside wig provision. To date, there are no studies examining the effectiveness of psychological interventions specific to people living with alopecia; however, approaches such as cognitive behavioural therapy and compassion focused therapy may be beneficial in targeting processes underlying social anxiety. Attentional training techniques such as mindfulness may also be beneficial to reduce pre-occupation with appearance related thoughts [30].

Acknowledgements

The authors would like to thank alopecia UK for their support in recruitment for this study.

Contributors AT and KM developed the protocol for this study. All authors have contributed to the preparation of this manuscript. KM carried out data analysis for

For peer review only - http://bmjopen.bmj.com/site/about/guidelines.xhtml

quantitative data and KM and CW analysed qualitative data to ensure reliability of coding. All data was checked by AT.

Funding This work was supported by the Economic and Social Research Council Grant number ES/J00215/1

Competing interests None declared.

Ethics approval Department of Psychology ethics committee, University of Sheffield

Data sharing statement The protocol and data files are available from the corresponding author on request.

For peer review only

References

1. Thompson AR. Skin conditions. *Encyclopaedia of Body Image and Human Appearance*; 738-744.

2. Tucker P. Bald is beautiful? The psychosocial impact of alopecia areata. J Health Psychol. 2009;**14**:142-51. doi: 10.1177/1359105308097954

3. Hunt N, McHale SU. Reported experiences of persons with alopecia areata. Journal of Loss and Trauma. 2004;**10**:33-50. doi: 10.1080/15325020490890633

4. Hunt N, McHale S. The psychological impact of alopecia. *BMJ*. 2005; **20**;7522:951.

5. Rumsey N, Harcourt D. Body image and disfigurement: issues and interventions. *Body image*. 2004;**1**(1):83-97. doi: 10.1016/S1740-1445(03)00005-6

6. Thompson A, Kent G. Adjusting to disfigurement: processes involved in dealing with being visibly different. *Clin Psychol Rev*. 2001;**21**(5):663-82. doi: 10.1016/S0272-7358(00)00056-8

7. Ruiz-Doblado S, Carrizosa A, García-Hernández MJ. Alopecia areata: psychiatric comorbidity and adjustment to illness. *Int J Dermatol*. 2003;**42**(6):434-7. doi: 10.1046/j.1365-4362.2003.01340.x

8. Koo JY, Shellow WV, Hallman CP, Edwards JE. Alopecia areata and increased prevalence of psychiatric disorders. *Int J Dermatol*. 1994;**33**(12):849-50. doi: 10.1111/j.1365-4362.1994.tb01018.x

9. Cash TF. The psychological effects of androgenetic alopecia in men. *J Am. Acad. Dermatol*. 1992;**26**(6):926-31. doi: 10.1016/0190-9622(92)70134-2

10. Cash TF, Price VH, Savin RC. Psychological effects of androgenetic alopecia on women: comparisons with balding men and with female control subjects. *J Am. Acad. Dermatol*. 1993;**29**(4):568-75. doi: 10.1016/0190-9622(93)70223-G

11. Chiang YZ, Bundy C, Griffiths CE, Paus R, Harries MJ. The role of beliefs: lessons from a pilot study on illness perception, psychological distress and quality of life in patients with primary cicatricial alopecia. *Brit J Dermatol*. 2015;**172**(1):130-7. doi: 10.1111/bjd.13259

12. Messenger AG, McKillop J, Farrant P, McDonagh AJ, Sladden M. British Association of Dermatologists' guidelines for the management of alopecia areata 2012. *Brit J Dermatol*. 2012;**166**(5):916-26. doi: 10.1111/j.1365-2133.2012.10955.x

13. Wiggins S, Moore-Millar K, Thomson A. Can you pull it off? Appearance modifying behaviours adopted by wig users with alopecia in social interactions. *Body image*. 2014;**11**(2):156-66. doi: 10.1016/j.bodyim.2014.01.004

14. Welsh N, Guy A. The lived experience of alopecia areata: a qualitative study. *Body Image*. 2009;**6**(3):194-200. 10.1016/j.bodyim.2009.03.004

15. Cash TF. The psychology of hair loss and its implications for patient care. *Clin Dermatol.* 2001;**19**(2):161-6. doi: 10.1016/S0738-081X(00)00127-9

For peer review only

16. Goffman E. *Stigma: Notes on the management of spoiled identity*. Simon and Schuster. 2009.

17. Kacar SD, Soyucok E, Bagcioglu E, Ozuguz P, Coskun KS, Asık AH, Mayda H. The perceived stigma in patients with alopecia and mental disorder: A comparative study. *Int J Trichology*. 2016;**8**(3):135. doi: 10.4103/0974-7753.189005

18. Bewley A, Affleck A, Bundy C, Higgins E, McBride S. Psychodermatology services guidance: The report of the British Association of Dermatologists' Psychodermatology Working Party. *Brit J Dermatol*. 2013;**168**:1149-50. doi: 10.1111/bjd.12330

19. Lavda AC, Webb T, Thompson AR. A meta-analysis of the effectiveness of psychological interventions for adults with skin conditions. *Brit J Dermatol*. 2012;**167**:970-9. doi: 10.1111/j.1365-2133.2012.11183.x

20. Macbeth AE, Tomlinson J, Messenger AG, Moore-Millar K, Michaelides C, Shipman AR, Kassim JM, Brockley JR, Szczecinska W, Farrant P, Robinson R. Establishing and Prioritising Research Questions for the Treatment of Alopecia Areata: The Alopecia Areata Priority Setting Partnership. *Brit J Dermatol*. 2016; Oct 1. doi: 10.1111/bjd.15099

21. Connor KM, Davidson JR, Churchill LE, Sherwood A, Weisler RH, Foa E. Psychometric properties of the social phobia inventory (SPIN). *Brit J Psychiat*. 2000;**176**(4):379-86. doi: 10.1192/bjp.176.4.379

22. Spitzer RL, Kroenke K, Williams JB, Patient Health Questionnaire Primary Care Study Group. Validation and utility of a self-report version of PRIME-MD: the PHQ primary care study. *Jama.* 1999;**282**(18):1737-44. doi: 10.1001/jama.282.18.1737

23. Kroenke K, Spitzer RL, Williams JB. The Phq-9. *J Gen Intern Med*. 2001;**16**(9):606-13. doi: 10.1046/j.1525-1497.2001.016009606.x

24. Spitzer RL, Kroenke K, Williams JB, Löwe B. A brief measure for assessing generalized anxiety disorder: the GAD-7. *Archives of Internal Medicine.* 2006;**166**(10):1092-7. doi: /10.1001archinte.166.10.1092

25. Kroenke K, Spitzer RL, Williams JBW, Monahan PO, Löwe B. Anxiety disorders in primary care: prevalence, impairment, comorbidity, and detection. *Ann Intern Med*. 2007;**146**(5):317–25. doi: 10.7326/0003-4819-146-5-200703060-00004

26.

27. Mayring P. Qualitative content analysis. *Qualitative Social Research*. 2000; **1**(2). Available from: www.qualitative-research.net/index.php/fqs/article/view/1089/2385 (Accessed 29th August 2016)

28. Rosman S. Cancer and stigma: experience of patients with chemotherapy-induced alopecia. *Patient Edu Couns*. 2004;**52**(3):333-9. doi: 10.1016/S0738-3991(03)00040-5

For peer review only

29. Smart Richman L, Leary MR. Reactions to discrimination, stigmatization, ostracism, and other forms of interpersonal rejection: a multimotive model. Psychol Rev. 2009;**116**(2):365. doi: 10.1037/a0015250

30. Clarke A, Thompson AR, Jenkinson E, Rumsey N, Newell R. *CBT for appearance anxiety: psychosocial interventions for anxiety due to visible difference*. John Wiley & Sons. 2013.

For peer review only

Table 1. Participant characteristics

		N	%
Type of alopecia	Alopecia areata	114	33.7
	Universalis	106	31.4
	Totalis	59	17.5
	Frontal fibrosing	7	2.1
	Androgenic	8	2.4
	Male patterned baldness	3	0.9
	Scarring	3	0.9
	Female patterned baldness	2	0.6
	Lichen planopilaris	1	0.3
	Folliculitis decalvaris	1	0.3
	Chemically induced	1	0.3
	Unknown	32	9.5
Gender	Female	329	97.3
	Male	5	1.5
	No response	1	0.3
Ethnicity	White Caucasian	316	93.5
	Asian/ British Asian	10	3.0
	Black/ Black British	5	1.5
	Other	4	1.2
	No response	1	0.3

For peer review only

Table 2. Wig use of participants (N = 338)

How often do you wear a wig	% of people wearing a wig
Never	23.6%
Occasionally	16.6%
Most of the time	26%
All the time (excluding the night)	44.1%
All the time (including the night)	5.9%

Table 3. Situations in which people would wear a wig (N = 338)

Situations when you would wear a wig	% of people wearing a wig
Meeting new people	78.4
Dating	61.2
Socialising	86.7
Work/school/university	76.3
All the time	55.9

STROBE Statement

	Item No	Recommendation	Page Number
**Title and abstract**	1	(*a*) Indicate the study's design with a commonly used term in the title or the abstract	1
		(*b*) Provide in the abstract an informative and balanced summary of what was done and what was found	2
**Introduction**			
Background/rationale	2	Explain the scientific background and rationale for the investigation being reported	4-5
Objectives	3	State specific objectives, including any prespecified hypotheses	4-5
**Methods**			
Study design	4	Present key elements of study design early in the paper	5
Setting	5	Describe the setting, locations, and relevant dates, including periods of recruitment, exposure, follow-up, and data collection	5
Participants	6	(*a*) *Cohort study*—Give the eligibility criteria, and the sources and methods of selection of participants. Describe methods of follow-up<br>*Case-control study*—Give the eligibility criteria, and the sources and methods of case ascertainment and control selection. Give the rationale for the choice of cases and controls<br>*Cross-sectional study*—Give the eligibility criteria, and the sources and methods of selection of participants	Cross-sectional study 5 (paragraph 1) further information on participant characteristics on p. 6
		(*b*) *Cohort study*—For matched studies, give matching criteria and number of exposed and unexposed<br>*Case-control study*—For matched studies, give matching criteria and the number of controls per case	
Variables	7	Clearly define all outcomes, exposures, predictors, potential confounders, and effect modifiers. Give diagnostic criteria, if applicable	7
Data sources/ measurement	8*	For each variable of interest, give sources of data and details of methods of assessment (measurement). Describe comparability of assessment methods if there is more than one group	7
Bias	9	Describe any efforts to address potential sources of bias	Cross-sectional study. Recruitment via charity discussed in limitations p.15
Study size	10	Explain how the study size was arrived at	6

For peer review only - http://bmjopen.bmj.com/site/about/guidelines.xhtml

Quantitative variables	11	Explain how quantitative variables were handled in the analyses. If applicable, describe which groupings were chosen and why	5-6
Statistical methods	12	(*a*) Describe all statistical methods, including those used to control for confounding	5-6
		(*b*) Describe any methods used to examine subgroups and interactions	n/a
		(*c*) Explain how missing data were addressed	n/a
		(*d*) *Cohort study*—If applicable, explain how loss to follow-up was addressed<br>*Case-control study*—If applicable, explain how matching of cases and controls was addressed<br>*Cross-sectional study*—If applicable, describe analytical methods taking account of sampling strategy	All responses complete
		(*e*) Describe any sensitivity analyses	n/a

Continued on next page

For peer review only

Results			Page
Participants	13*	(a) Report numbers of individuals at each stage of study—eg numbers potentially eligible, examined for eligibility, confirmed eligible, included in the study, completing follow-up, and analysed	6
		(b) Give reasons for non-participation at each stage	n/a
		(c) Consider use of a flow diagram	n/a
Descriptive data	14*	(a) Give characteristics of study participants (eg demographic, clinical, social) and information on exposures and potential confounders	6
		(b) Indicate number of participants with missing data for each variable of interest	n/a
		(c) *Cohort study*—Summarise follow-up time (eg, average and total amount)	n/a
Outcome data	15*	*Cohort study*—Report numbers of outcome events or summary measures over time	n/a
		Case-control study—Report numbers in each exposure category, or summary measures of exposure	n/a
		Cross-sectional study—Report numbers of outcome events or summary measures	6-7
Main results	16	(*a*) Give unadjusted estimates and, if applicable, confounder-adjusted estimates and their precision (eg, 95% confidence interval). Make clear which confounders were adjusted for and why they were included	n/a
		(*b*) Report category boundaries when continuous variables were categorized	7
		(*c*) If relevant, consider translating estimates of relative risk into absolute risk for a meaningful time period	n/a
Other analyses	17	Report other analyses done—eg analyses of subgroups and interactions, and sensitivity analyses	n/a
**Discussion**			
Key results	18	Summarise key results with reference to study objectives	13-15
Limitations	19	Discuss limitations of the study, taking into account sources of potential bias or imprecision. Discuss both direction and magnitude of any potential bias	15
Interpretation	20	Give a cautious overall interpretation of results considering objectives, limitations, multiplicity of analyses, results from similar studies, and other relevant evidence	16
Generalisability	21	Discuss the generalisability (external validity) of the study results	15
**Other information**			
Funding	22	Give the source of funding and the role of the funders for the present study and, if applicable, for the original study on which the present article is based	n/a

*Give information separately for cases and controls in case-control studies and, if applicable, for exposed and unexposed groups in cohort and cross-sectional studies.

Note: An Explanation and Elaboration article discusses each checklist item and gives methodological background and published examples of transparent reporting. The STROBE checklist is best used in conjunction with this article (freely available on the Web sites of PLoS Medicine at

For peer review only

http://www.plosmedicine.org/, Annals of Internal Medicine at http://www.annals.org/, and Epidemiology at http://www.epidem.com/). Information on the STROBE Initiative is available at www.strobe-statement.org.

For peer review only

For peer review only - http://bmjopen.bmj.com/site/about/guidelines.xhtml

BMJ Open

A mixed methods survey of social anxiety, anxiety, depression, and wig use in alopecia.

Journal:	*BMJ Open*
Manuscript ID	bmjopen-2016-015468.R2
Article Type:	Research
Date Submitted by the Author:	21-Mar-2017
Complete List of Authors:	Montgomery, Kerry; University of Sheffield, Psychology<br>White, Caroline; University of Manchester, Dermatopharmacology Unit<br>Thompson, Andrew; University of Sheffield, Department of Psychology
**Primary Subject Heading**:	Dermatology
Secondary Subject Heading:	Health services research, Mental health, Public health, Qualitative research
Keywords:	Psychodermatology, wig provision, alopecia

SCHOLARONE™
Manuscripts

For peer review only

A mixed methods survey of social anxiety, anxiety, depression, and wig use in alopecia.

K. Montogmery[1]., C. White[2]., & A.R. Thompson[1]

[1] Department of Psychology, University of Sheffield

[2] Caroline White RN, Research Nurse, University of Manchester, Faculty of Biology, Medicine and Health, Dermatopharmacology Unit. Telephone: 0161 206 0893. Email: caroline.white@manchester.ac.uk

Corresponding author: Kerry Montgomery, Department of Psychology, University of Sheffield, Cathedral Court, 1 Vicar Lane, Sheffield, S1 2LT. UK. Tel: +44 (0)114 2226637. Email: kmontgomery1@sheffield.ac.uk

Conflict of interest disclosure: The authors have no conflicts of interest to disclose

For peer review only

Abstract

Objectives: This study aimed to examine levels of social anxiety, anxiety and depression reported by people with alopecia as a result of a dermatological condition, and associations with wig use. The study also sought to report on experiences of wearing wigs in social situations and the relationship with social confidence.

Design: A cross sectional survey was sent by email to the Alopecia UK charity mailing list and advertised on social media.

Participants: Inclusion criteria were a diagnosis of alopecia, aged thirteen or above and sufficient English to complete the survey. Exclusion criteria included; experiencing hair loss as a result of chemotherapy treatment or psychological disorder. Participants (N=338) were predominantly female (97.3%), Caucasian (93.5%), aged between 35-54 years (49.4%) with a diagnosis of alopecia areata (82.6%).

Main outcome measures: The Social Phobia Inventory measured symptoms of social anxiety, and the Hospital Anxiety and Depression Scale was used to measure symptoms of anxiety and depression. Survey questions were designed to measure the use of wigs. Open-ended questions enabled participants to comment on experiences of wearing wigs.

Results: Clinically significant levels of social anxiety (47.5%), anxiety (35.5%) and depression (29%) were reported. Participants who reported worries about not wearing a wig reported significantly higher levels of depression, $t(103) = 3.40$, $p = <.001$, anxiety, $t(109) = 4.80$, $p = <.001$, and social anxiety, $t(294)$, $= 3.89$, $p = <.001$. Wearing wigs was reported as increasing social confidence; however, the concealment it afforded was also reported as both reducing fear of negative evaluation, but also maintaining anxiety.

Discussion: Overall 46% of participants reported that wearing a wig had a positive impact on their everyday life with negative experiences related to fears of the wig being

For peer review only

noticed. Psychological interventions alongside wig provision would be beneficial for people living with alopecia.

Strengths and limitations of this study

- This is the first study to examine National Health Service wig provision and how people living with alopecia finance wigs.
- Validated measures of social anxiety, anxiety and depression were used to determine levels of distress.
- Survey questions investigated the experience of wearing wigs.
- The sample was predominantly female and Caucasian therefore further research is needed to examine experiences of men and cultural differences in alopecia.
- Participants recruited from Alopecia UK may have been experiencing higher levels of distress than the general population having accessed support by the charity.

For peer review only

Introduction

For many people hair is a central aspect of appearance and self-image therefore hair loss can have a negative impact on self-esteem, body image and confidence [1-2]. Whilst hair loss may have few physical health consequences living with a condition that affects appearance can have a significant impact on every day functioning [3-6]. Studies have shown that people living with alopecia are at higher risk of developing depression, anxiety and social phobia than the general population [7-8].

Higher levels of body dissatisfaction and preoccupation with appearance have been reported in men and women living with alopecia, relative to controls [7, 9-10], with 40% of women reporting marital problems as a consequence [3]. Perception of hair loss has a significant impact on quality of life, accounting for 35% of the variance [11].

Wearing a wig to conceal hair loss is an important coping strategy for many individuals [12-15] giving people the confidence to return to work and engage in social activity [14]. We have known since the time of Goffman's seminal work on stigmatisation [16] that people may choose to conceal attributes they believe will be discredited by others. Recent studies indicate that alopecia areata can be associated with higher levels of perceived stigmatisation than concealable conditions such as mental health conditions [17]. Wearing a wig might reduce an individuals' perception of a discrepancy between cultural norms regarding the appearance of the hair and their own appearance. However, wearing a wig might not be an ideal solution as the fear of being 'discredited' should the wig be noticed remains [16].

Despite wigs being an important coping strategy for people living with alopecia many people experience difficulties accessing wig prescriptions via the National Health Service (NHS). Some NHS trusts report that alopecia is a 'cosmetic issue' therefore the financial

For peer review only

burden of wigs falls with the patient. Further cuts in dermatology could mean more patients have difficulties accessing wigs which may have significant psychosocial consequences for people living with alopecia. Access to psychological support within dermatology is limited [18] and currently there are no psychological interventions available to target the specific needs of this population [19]. The need for psychological interventions for alopecia has been emphasised by patients and clinicians [20].

The current mixed methods study aims to examine (i) levels of social anxiety, anxiety and depression reported by people living with alopecia, (ii) associations between wig behaviours and psychosocial distress, and (iii) experiences of wearing wigs in social situations. The current study will also investigate how participants access wigs and the financial implications of wearing wigs.

Method

A cross sectional survey incorporating validated measures of social anxiety, depression and anxiety, questions on wig usage, financing wigs, and open ended questions regarding experiences of wearing wigs in social situations was sent by email to the Alopecia UK mailing list, and advertised on social media. Ethical approval for the study was gained from the University of Sheffield Psychology department ethics committee. Inclusion criteria were: a diagnosis of alopecia as a result of a dermatological condition, aged thirteen or above and sufficient English to complete the survey. Participants aged under 16 years of age were advised to complete the questionnaire with a parent or guardian. Exclusion criteria included; experiencing hair loss as a result of chemotherapy treatment or primary psychological disorder (e.g., trichotillomania).

Measures

Social anxiety

For peer review only

The Social phobia inventory (SPIN) [21] is a 17 item self-report questionnaire to measure various elements of social anxiety (Fear in social situations, avoidance of social situations/ performances and physiological symptoms in social situations). Connor et al [21] reported a mean score of 41 on the SPIN for individuals with social phobia, compared to a score of 12 in a non-psychiatric population. A score of 19 or above indicates symptoms of social anxiety with 79% diagnostic accuracy [21].

Depression

The 9-item self-report Patient Health Questionnaire [22] was used to measure symptoms of depression experienced over the last two weeks. A mean score of three has been reported by healthy controls, in comparison to a score of 17 in people diagnosed with depression [22]. A cut-off score of ≥ 10 was used to identify individuals experiencing depression (88% specificity 88% sensitivity) [23].

Anxiety

The Generalised Anxiety Disorder Questionnaire [24] is a 7 item self-report scale to measure generalized anxiety symptoms over the last two weeks. A mean score of 4.9 has been reported in healthy controls, in comparison to a score of 14 in people living with generalised anxiety [24]. A cut off score of ≥ 8 was used to identify individuals experiencing depression [25].

Survey questions

A number of survey questions were included in which participants were asked to report on the situations in which they would choose to wear a wig (e.g., social situations, dating, work/university/school), and if they had any worries about financing or not wearing wigs. Open ended questions were asked to elicit experiences of wearing wigs in social

For peer review only

situations. Questions included: Has wearing a wig had any impact on your confidence during social situations? Do you have any worries about not wearing a wig? and, how has wearing a wig affected your everyday life?

Analysis

Statistical analysis was carried out using SPSS version 22 [26]. Descriptive statistics were used to provide information on (i) demographic and clinical variables, (ii) percentage of people wearing wigs and the situations in which people would choose to wear wigs, (iii) wig funding and (iii) clinically significant levels of social anxiety, depression and anxiety. Associations were tested between demographic variables, wig behaviour (e.g., situations in which people would choose to wear wigs) and social anxiety, depression and anxiety using independent samples t tests and Pearson correlation as appropriate. An alpha level of .05 was used for all statistical tests.

Qualitative responses were analysed using qualitative content analysis [27]. QCA is a systematic text analysis technique which preserves the advantages of quantitative analysis, allowing frequencies of data to be reported whilst also providing a method of interpreting participant experiences of wearing wigs during social situations [27].

An inductive approach to data analysis was used to determine emergent themes generated through examination of participant’s responses to specific questions. The data was analysed based on the wording used by participants to describe their experiences, and organised into higher order themes. For example, the response that wigs ‘improved confidence’ and participants ‘felt more able to go out’, was coded into the themes ‘improved confidence’ and ‘positive experience of a wearing wig’. Frequency of the occurrence of themes across participants was recorded by the first and second author.

For peer review only

Inter-rater reliability (98%) was established by determining any differences in coding between the first and second authors.

Results

Participants

Participants (N =338) were predominantly female (97.3%) and Caucasian (93.5%). All participants lived in the UK. The majority of the sample were aged between 35-54 years (49.4%) (13-17 years = 1.5%, 18-25 years = 8%, 26-34 years = 13.6%, 55-64 years = 18.6%, 65 years and over = 8.9%). Eleven different types of alopecia were reported across the sample (Table 1) and the majority of the sample comprised of alopecia areata (82.6%). Of the respondents 10.7% were receiving medical treatment for hair loss including steroid injections, spironolactone, and topical treatments.

The majority of participants chose to wear wigs most or all of the time (76%) (Table 2). The most common type of wigs worn by participants were acrylic (acrylic monofilament 39.3%, acrylic lace front 27.5% and acrylic wefted wig 14.8). The majority of participants did not obtain NHS wig prescriptions (50.6%) with 11.2% of participants been told they were not eligible and 22.5% had never enquired about it. Of participants who claimed NHS wig prescriptions (46.1%), 11.5% claimed one per year, 26 % claimed two per year and 8.6% claimed more than two each year.

Social anxiety, anxiety and depression

Clinically significant levels of anxiety (score ≥ 8) were reported by 35.5% of participants, with 40.5% reporting no symptoms, 14.5% reporting mild symptoms (score 7-9), 13.5% reporting moderate symptoms and 15% reporting severe symptoms of anxiety. Clinically significant levels of depression (score ≥ 10) were reported by 29% of the sample, with

For peer review only

43.6% reporting no symptoms, 27.6% reporting mild symptoms (score 5-9), 14.3% reporting moderate symptoms (score 10-14), 8.1% reporting moderately severe symptoms and 6.6% reporting severe symptoms of depression. Clinically significant symptoms of social anxiety (score ≥ 19) were reported by 47.5% of the sample, with 23.4% reporting mild symptoms, 11.1% reporting moderate symptoms, 7.2% reporting severe symptoms and 6.6% reporting very severe symptoms. Participants reporting worries about not wearing a wig reported significantly higher levels of depression, $t(103) = 3.40$, $p = <.001$, anxiety, $t(109) = 4.80$, $p = <.001$, and social anxiety, $t(294)$, $= 3.89$, $p = <.001$.

Wig use

Participants were asked when they wear a wig across a number of everyday activities (Table 3). The majority of participants reported wearing a wig to socialise (86.7%), and 66.3% of respondents reported they would not feel confident leaving the house without a wig. Of the participants who purchased wigs via NHS prescription, 28.1% reported that they would be unable to afford their wig privately and 65.1% of the sample reported worry about affording new wigs.

Qualitative findings

A summary of responses to open-ended survey responses is reported below.

Has wearing a wig had any impact on confidence during social situations?

Three hundred and thirteen participants responded to the question on the impact of wigs on confidence in social situations. Of responses, 26% reported a positive impact of wearing a wig. The positive impact was related to the reduced likelihood of comments

For peer review only

regarding hair loss in 23% of participants, and feeling more confident going out in public (32%).

> *“Interacting with people who I've never met before, without a wig they may wonder why I have no hair, it is not, the norm to have patchy bald head. Hate the idea people will talk about me, or feel I have a more serious illness”*

> *“At different times in my life a wig has been a survival tool for me”*

Participants also reported that wearing a wig could have a negative impact on confidence during social situations (43%). These responses were categorised into two main themes: worries about others knowing it was a wig (47%) and concerns about the wig coming off/ discomfort (39%). Wearing a wig also led to reduced activity (41%) in particular sports were avoided due to concerns about having to take off the wig.

> *“Even 10 years on, I still wonder if people can tell I wear a wig and especially when I meet new people. It has stopped me going to places or trying new activities”*

Difficulty adjusting to a change in appearance when wearing a wig was reported with participants describing feeling disconnected from their appearance in a wig.

> *“No matter how attractive I look in a wig I feel like a sham. I feel the real bald me is not attractive at all”*

For some participants (7.8%) wearing a wig had no impact on their confidence, which was due to positive experiences of wearing a wig. Other responses were not coded as participants had simply responded ‘no’ which was interpreted as ‘no impact’ but it was unclear if this was related to a positive or negative impact of wigs.

For peer review only

How has wearing a wig affected your everyday life?

Of two hundred and ninety-nine responses, 23% of participants reported that wearing a wig improved their confidence/self-esteem in everyday life.

> *"It's improved my confidence as I avoided harsh overhead lights in retail stores as it emphasised my thinning hair particularly distressing when I saw my reflection in a mirror. Now I can walk past a mirror with harsh lighting overhead and check my hair and smile!"*

> *"It's given me a lot more confidence in just leaving the house and being in front of other people."*

Negative views of appearance without a wig were reported (14%).

> *"Don't like anyone seeing me without it, feel naked."*

> *"I feel a lot less feminine without hair"*

Participants reported negative reactions from others if they were not wearing a wig (9.6%) suggesting wigs were used as a coping strategy to manage negative reactions.

> *"It is an uncomfortable but manageable part of my life. It means that I don't get as many pitying glances from strangers."*

> *"I feel more confident to go out with my children, it becomes incredibly tiring been stared at and shouted at if I ever left the house without my wig on."*

Overall, 46.6% of participants reported that wearing a wig had a positive impact on their everyday life, reducing the likelihood of negative reactions whilst improving confidence in regards to appearance.

For peer review only

Participants also reported problems with wigs which had a negative impact on everyday life (49%; subthemes included: wigs coming off, expense, quality and fitting).

> *"Wearing a wig is a way of life to me as I've never had hair, but always dreamed of being normal. Not worrying if it's going to fall off or move"*

> *"Every time I step outside my front door I think about weather conditions, who I might meet, if anyone is going to stand too close to me, what they will see, what they will think."*

In addition, wearing wigs reduced social activity (33%) in particular, dating, meeting new people and exercise were situations in which participants experienced problems.

> *"It's depressing every morning having to wake up and put my wig on. When I sleep with someone new in my room I don't take my wig off "*

> *"Constant worry that someone/child will randomly pull the hair, or know it is a wig and make comments. Still anxious when out, but at least I now leave the house."*

> *"Going swimming is difficult I don't like swimming hats, and use an old wig, but can't dive in a pool or swim under water with a wig on, constantly scared of losing wig in water, but love to swim."*

Participants reported feeling more self-conscious about having to wear a wig (17%). Self-consciousness appeared to arise as a result of concerns about others finding out they were wearing a wig (22%).

> "The NHS wigs look like wigs so I always imagine people are saying "Look at her wearing that stupid wig ". I only go out if absolutely necessary and avoid public places as often as I can. I FEEL LIKE A FREAK"

For peer review only

"I've had bad experiences at work and socially where someone took my wig for a bet/ cos they were drunk"

Worries about not wearing a wig

Participants reported worries about not wearing a wig (40.5%) due to the reactions of others to hair loss (staring 42%, comments 18%).

"People as a rule have hair, why would I want people to stare and comment?"

Specific concerns about not wearing a wig included worries others would assume they had cancer (n = 13).

"People assume I have cancer. I look strange"

Self-conscious emotions and negative self-appraisals of appearance also led to worries about not wearing a wig (19%).

"I am worried people will see how ugly I really am if they see me bald"

Discussion

The first aim of the current study was to investigate the prevalence of social anxiety, anxiety and depression in people living with alopecia. Social anxiety was commonly reported by the participants in this study with 47.5% indicating clinically significant levels of social anxiety. Whilst few quantitative studies have examined the prevalence of social anxiety in people living with alopecia specifically; qualitative studies suggest that alopecia can have a significant impact on social functioning [2-4, 13-14]. The prevalence of anxiety and depression reported are consistent with previous studies [2], and highlights the importance of identifying coping strategies to help people living with alopecia to manage psychological distress.

For peer review only

The current study also aimed to examine the relationship between psychological variables and wig usage. Worry about not wearing a wig was related to higher levels of depression, anxiety and social anxiety and with 65% of participants reporting worry about affording new wigs, it suggests the financial burden of affording wigs could lead to additional distress in patients already reporting significant levels of social anxiety, depression and anxiety. This finding has important implications in the current economic climate and suggests changes in NHS wig provision could have negative psychosocial consequences should people with alopecia no longer be able to access wig prescriptions.

For some people living with alopecia, this study clearly shows that wearing a wig is an important coping strategy, helping people to manage negative reactions from others and improving confidence to engage in social activity. However, despite wigs being viewed as an important coping strategy only 46.7% of participants who chose to wear wigs accessed NHS wig prescriptions, with some NHS trusts failing to provide any wig provision for patients with alopecia.

The final aim of the current study was to explore participants experiences of wearing wigs in social situations. The current findings suggest that the relationship between wigs and social confidence is complex and wigs alone do not appear to reduce social distress for a large number of patients. The current findings indicate that people living with alopecia face a difficult choice in social situations, negative reactions as a result of hair loss being visible, or negative reactions a result of wearing a wig. Managing the noticeability of wigs appeared to lead to significant negative interpersonal consequences, including avoidance of social situations and exercise. Despite generating anxiety and causing discomfort, participants were still choosing to wear wigs suggesting wigs were seen as the 'lesser of two evils' given worries about reactions of others to hair loss.

For peer review only

These findings are consistent with previous studies and suggest that wearing a wig does not signify an end point to managing social distress for people living with alopecia[13-14].

Critical self-appraisals of appearance without a wig ('freak, alien, ugly') appeared to be a contributing factor to wearing a wig, as wigs improved confidence in social situations through reducing appearance concern. Hair loss was viewed as a negative attribute with participants reporting experiences of stigma including comments and staring, and perception of hair loss as a sign of illness. Hair loss is reported as confirmation of identity for cancer patients [28] which has social implications for people living with alopecia, in that they are often mis-identified as 'ill'.

Negative reactions from others represent threats to an individual's desire to be valued and accepted by others [16]. Reactions to social rejection may be prosocial, involving an increased desire to be socially accepted; antisocial, whereby the individual attempts to defend themselves or express anger following rejection, and avoidant [29], in which an individual may seek to avoid further rejection which could involve reduced social engagement. Wearing a wig may therefore be seen as a strategy to increase social acceptance and to avoid rejection. Indeed, Goffman's seminal work on stigma [16] suggests that individuals may choose to conceal attributes they believe will be discredited by others, in this case hair loss; however, the consequence of this is continued concealment and anxiety about being 'found out'.

There are several limitations of the current study which should be noted. Firstly, the sample is predominantly female and Caucasian therefore further research is needed to explore the experiences of men and cultural differences in experiences of alopecia. Second, a number of responses could not be coded into themes as the responses did not relate to the question suggesting some participants may have experienced problems

For peer review only

interpreting the questions. Third, participants were recruited from Alopecia UK, therefore some participants may have been experiencing higher levels of distress than the general population having accessed support via the charity. Finally, given that participants were recruited from the Alopecia UK mailing list and social media it is not possible to ascertain a response rate in the current study.

The current findings have important implications when providing support to people living with alopecia and suggest that wigs appear to be much more than a ‘cosmetic’ tool, and are used to reduce potential experiences of stigmatisation and improve social confidence. Given the significant negative social consequences that could arise should wigs no longer be an option, the current findings should be considered when funding decisions are made regarding wig provision for patients with alopecia. Negative experiences of wearing wigs are related to fears of the wig being noticed and hair loss being discovered; therefore, psychological interventions, particularly those targeting self-conscious emotions such as shame and social anxiety would be beneficial for people living with alopecia alongside wig provision. To date, there are no studies examining the effectiveness of psychological interventions specific to people living with alopecia; however, approaches such as cognitive behavioural therapy and compassion focused therapy may be beneficial in targeting processes underlying social anxiety. Attentional training techniques such as mindfulness may also be beneficial to reduce pre-occupation with appearance related thoughts [30].

Acknowledgements

The authors would like to thank alopecia UK for their support in recruitment for this study.

Contributors AT and KM developed the protocol for this study. All authors have contributed to the preparation of this manuscript. KM carried out data analysis for

For peer review only

quantitative data and KM and CW analysed qualitative data to ensure reliability of coding. All data was checked by AT.

Funding This work was supported by the Economic and Social Research Council Grant number ES/J00215/1

Competing interests None declared.

Ethics approval Department of Psychology ethics committee, University of Sheffield

Data sharing statement The protocol and data files are available from the corresponding author on request.

For peer review only

References

1. Thompson AR. Skin conditions. *Encyclopaedia of Body Image and Human Appearance*; 738-744.

2. Tucker P. Bald is beautiful? The psychosocial impact of alopecia areata. J Health Psychol. 2009;**14**:142-51. doi: 10.1177/1359105308097954

3. Hunt N, McHale SU. Reported experiences of persons with alopecia areata. Journal of Loss and Trauma. 2004;**10**:33-50. doi: 10.1080/15325020490890633

4. Hunt N, McHale S. The psychological impact of alopecia. *BMJ*. 2005; **20**;7522:951.

5. Rumsey N, Harcourt D. Body image and disfigurement: issues and interventions. *Body image*. 2004;**1**(1):83-97. doi: 10.1016/S1740-1445(03)00005-6

6. Thompson A, Kent G. Adjusting to disfigurement: processes involved in dealing with being visibly different. *Clin Psychol Rev*. 2001;**21**(5):663-82. doi: 10.1016/S0272-7358(00)00056-8

7. Ruiz-Doblado S, Carrizosa A, García-Hernández MJ. Alopecia areata: psychiatric comorbidity and adjustment to illness. *Int J Dermatol*. 2003;**42**(6):434-7. doi: 10.1046/j.1365-4362.2003.01340.x

8. Koo JY, Shellow WV, Hallman CP, Edwards JE. Alopecia areata and increased prevalence of psychiatric disorders. *Int J Dermatol*. 1994;**33**(12):849-50. doi: 10.1111/j.1365-4362.1994.tb01018.x

9. Cash TF. The psychological effects of androgenetic alopecia in men. *J Am. Acad. Dermatol*. 1992;**26**(6):926-31. doi: 10.1016/0190-9622(92)70134-2

10. Cash TF, Price VH, Savin RC. Psychological effects of androgenetic alopecia on women: comparisons with balding men and with female control subjects. *J Am. Acad. Dermatol*. 1993;**29**(4):568-75. doi: 10.1016/0190-9622(93)70223-G

11. Chiang YZ, Bundy C, Griffiths CE, Paus R, Harries MJ. The role of beliefs: lessons from a pilot study on illness perception, psychological distress and quality of life in patients with primary cicatricial alopecia. *Brit J Dermatol*. 2015;**172**(1):130-7. doi: 10.1111/bjd.13259

12. Messenger AG, McKillop J, Farrant P, McDonagh AJ, Sladden M. British Association of Dermatologists' guidelines for the management of alopecia areata 2012. *Brit J Dermatol*. 2012;**166**(5):916-26. doi: 10.1111/j.1365-2133.2012.10955.x

13. Wiggins S, Moore-Millar K, Thomson A. Can you pull it off? Appearance modifying behaviours adopted by wig users with alopecia in social interactions. *Body image*. 2014;**11**(2):156-66. doi: 10.1016/j.bodyim.2014.01.004

14. Welsh N, Guy A. The lived experience of alopecia areata: a qualitative study. *Body Image*. 2009;**6**(3):194-200. 10.1016/j.bodyim.2009.03.004

15. Cash TF. The psychology of hair loss and its implications for patient care. *Clin Dermatol.* 2001;**19**(2):161-6. doi: 10.1016/S0738-081X(00)00127-9

For peer review only

16. Goffman E. *Stigma: Notes on the management of spoiled identity*. Simon and Schuster. 2009.

17. Kacar SD, Soyucok E, Bagcioglu E, Ozuguz P, Coskun KS, Asık AH, Mayda H. The perceived stigma in patients with alopecia and mental disorder: A comparative study. *Int J Trichology*. 2016;**8**(3):135. doi: 10.4103/0974-7753.189005

18. Bewley A, Affleck A, Bundy C, Higgins E, McBride S. Psychodermatology services guidance: The report of the British Association of Dermatologists' Psychodermatology Working Party. *Brit J Dermatol*. 2013;**168**:1149-50. doi: 10.1111/bjd.12330

19. Lavda AC, Webb T, Thompson AR. A meta-analysis of the effectiveness of psychological interventions for adults with skin conditions. *Brit J Dermatol*. 2012;**167**:970-9. doi: 10.1111/j.1365-2133.2012.11183.x

20. Macbeth AE, Tomlinson J, Messenger AG, Moore-Millar K, Michaelides C, Shipman AR, Kassim JM, Brockley JR, Szczecinska W, Farrant P, Robinson R. Establishing and Prioritising Research Questions for the Treatment of Alopecia Areata: The Alopecia Areata Priority Setting Partnership. *Brit J Dermatol*. 2016; Oct 1. doi: 10.1111/bjd.15099

21. Connor KM, Davidson JR, Churchill LE, Sherwood A, Weisler RH, Foa E. Psychometric properties of the social phobia inventory (SPIN). *Brit J Psychiat*. 2000;**176**(4):379-86. doi: 10.1192/bjp.176.4.379

22. Spitzer RL, Kroenke K, Williams JB, Patient Health Questionnaire Primary Care Study Group. Validation and utility of a self-report version of PRIME-MD: the PHQ primary care study. *Jama.* 1999;**282**(18):1737-44. doi: 10.1001/jama.282.18.1737

23. Kroenke K, Spitzer RL, Williams JB. The Phq-9. *J Gen Intern Med*. 2001;**16**(9):606-13. doi: 10.1046/j.1525-1497.2001.016009606.x

24. Spitzer RL, Kroenke K, Williams JB, Löwe B. A brief measure for assessing generalized anxiety disorder: the GAD-7*. Archives of Internal Medicine.* 2006;**166**(10):1092-7. doi: /10.1001archinte.166.10.1092

25. Kroenke K, Spitzer RL, Williams JBW, Monahan PO, Löwe B. Anxiety disorders in primary care: prevalence, impairment, comorbidity, and detection. *Ann Intern Med*. 2007;**146**(5):317–25. doi: 10.7326/0003-4819-146-5-200703060-00004

26.

27. Mayring P. Qualitative content analysis*. Qualitative Social Research*. 2000; **1**(2). Available from: www.qualitative-research.net/index.php/fqs/article/view/1089/2385 (Accessed 29th August 2016)

28. Rosman S. Cancer and stigma: experience of patients with chemotherapy-induced alopecia. *Patient Edu Couns*. 2004;**52**(3):333-9. doi: 10.1016/S0738-3991(03)00040-5

For peer review only

29. Smart Richman L, Leary MR. Reactions to discrimination, stigmatization, ostracism, and other forms of interpersonal rejection: a multimotive model. Psychol Rev. 2009;**116**(2):365. doi: 10.1037/a0015250

30. Clarke A, Thompson AR, Jenkinson E, Rumsey N, Newell R. *CBT for appearance anxiety: psychosocial interventions for anxiety due to visible difference*. John Wiley & Sons. 2013.

For peer review only

Table 1. Participant characteristics

		N	%
Type of alopecia	Alopecia areata	114	33.7
	Universalis	106	31.4
	Totalis	59	17.5
	Frontal fibrosing	7	2.1
	Androgenic	8	2.4
	Male patterned baldness	3	0.9
	Scarring	3	0.9
	Female patterned baldness	2	0.6
	Lichen planopilaris	1	0.3
	Folliculitis decalvaris	1	0.3
	Chemically induced	1	0.3
	Unknown	32	9.5
Gender	Female	329	97.3
	Male	5	1.5
	No response	1	0.3
Ethnicity	White Caucasian	316	93.5
	Asian/ British Asian	10	3.0
	Black/ Black British	5	1.5
	Other	4	1.2
	No response	1	0.3

For peer review only

Table 2. Wig use of participants (N = 338)

How often do you wear a wig	% of people wearing a wig
Never	23.6%
Occasionally	16.6%
Most of the time	26%
All the time (excluding the night)	44.1%
All the time (including the night)	5.9%

Table 3. Situations in which people would wear a wig (N = 338)

Situations when you would wear a wig	% of people wearing a wig
Meeting new people	78.4
Dating	61.2
Socialising	86.7
Work/school/university	76.3
All the time	55.9

For peer review only

STROBE Statement

	Item No	Recommendation	Page Number
**Title and abstract**	1	(*a*) Indicate the study's design with a commonly used term in the title or the abstract	1
		(*b*) Provide in the abstract an informative and balanced summary of what was done and what was found	2
**Introduction**			
Background/rationale	2	Explain the scientific background and rationale for the investigation being reported	4-5
Objectives	3	State specific objectives, including any prespecified hypotheses	4-5
**Methods**			
Study design	4	Present key elements of study design early in the paper	5
Setting	5	Describe the setting, locations, and relevant dates, including periods of recruitment, exposure, follow-up, and data collection	5
Participants	6	(*a*) *Cohort study*—Give the eligibility criteria, and the sources and methods of selection of participants. Describe methods of follow-up<br>*Case-control study*—Give the eligibility criteria, and the sources and methods of case ascertainment and control selection. Give the rationale for the choice of cases and controls<br>*Cross-sectional study*—Give the eligibility criteria, and the sources and methods of selection of participants	Cross-sectional study 5 (paragraph 1) further information on participant characteristics on p. 6
		(*b*) *Cohort study*—For matched studies, give matching criteria and number of exposed and unexposed<br>*Case-control study*—For matched studies, give matching criteria and the number of controls per case	
Variables	7	Clearly define all outcomes, exposures, predictors, potential confounders, and effect modifiers. Give diagnostic criteria, if applicable	7
Data sources/ measurement	8*	For each variable of interest, give sources of data and details of methods of assessment (measurement). Describe comparability of assessment methods if there is more than one group	7
Bias	9	Describe any efforts to address potential sources of bias	Cross-sectional study. Recruitment via charity discussed in limitations p.15
Study size	10	Explain how the study size was arrived at	6

For peer review only

Quantitative variables	11	Explain how quantitative variables were handled in the analyses. If applicable, describe which groupings were chosen and why	5-6
Statistical methods	12	(*a*) Describe all statistical methods, including those used to control for confounding	5-6
		(*b*) Describe any methods used to examine subgroups and interactions	n/a
		(*c*) Explain how missing data were addressed	n/a
		(*d*) *Cohort study*—If applicable, explain how loss to follow-up was addressed<br>*Case-control study*—If applicable, explain how matching of cases and controls was addressed<br>*Cross-sectional study*—If applicable, describe analytical methods taking account of sampling strategy	All responses complete
		(*e*) Describe any sensitivity analyses	n/a

Continued on next page

For peer review only

Results			Page
Participants	13*	(a) Report numbers of individuals at each stage of study—eg numbers potentially eligible, examined for eligibility, confirmed eligible, included in the study, completing follow-up, and analysed	6
		(b) Give reasons for non-participation at each stage	n/a
		(c) Consider use of a flow diagram	n/a
Descriptive data	14*	(a) Give characteristics of study participants (eg demographic, clinical, social) and information on exposures and potential confounders	6
		(b) Indicate number of participants with missing data for each variable of interest	n/a
		(c) *Cohort study*—Summarise follow-up time (eg, average and total amount)	n/a
Outcome data	15*	*Cohort study*—Report numbers of outcome events or summary measures over time	n/a
		Case-control study—Report numbers in each exposure category, or summary measures of exposure	n/a
		Cross-sectional study—Report numbers of outcome events or summary measures	6-7
Main results	16	(*a*) Give unadjusted estimates and, if applicable, confounder-adjusted estimates and their precision (eg, 95% confidence interval). Make clear which confounders were adjusted for and why they were included	n/a
		(*b*) Report category boundaries when continuous variables were categorized	7
		(*c*) If relevant, consider translating estimates of relative risk into absolute risk for a meaningful time period	n/a
Other analyses	17	Report other analyses done—eg analyses of subgroups and interactions, and sensitivity analyses	n/a
**Discussion**			
Key results	18	Summarise key results with reference to study objectives	13-15
Limitations	19	Discuss limitations of the study, taking into account sources of potential bias or imprecision. Discuss both direction and magnitude of any potential bias	15
Interpretation	20	Give a cautious overall interpretation of results considering objectives, limitations, multiplicity of analyses, results from similar studies, and other relevant evidence	16
Generalisability	21	Discuss the generalisability (external validity) of the study results	15
**Other information**			
Funding	22	Give the source of funding and the role of the funders for the present study and, if applicable, for the original study on which the present article is based	n/a

*Give information separately for cases and controls in case-control studies and, if applicable, for exposed and unexposed groups in cohort and cross-sectional studies.

Note: An Explanation and Elaboration article discusses each checklist item and gives methodological background and published examples of transparent reporting. The STROBE checklist is best used in conjunction with this article (freely available on the Web sites of PLoS Medicine at

For peer review only

http://www.plosmedicine.org/, Annals of Internal Medicine at http://www.annals.org/, and Epidemiology at http://www.epidem.com/). Information on the STROBE Initiative is available at www.strobe-statement.org.

For peer review only
